# Isolated cognitive impairment in people with multiple sclerosis: frequency, MRI patterns and its development over time

**DOI:** 10.1007/s00415-024-12185-8

**Published:** 2024-01-30

**Authors:** Piet M. Bouman, Maureen A. van Dam, Laura E. Jonkman, Martijn D. Steenwijk, Menno M. Schoonheim, Jeroen J. G. Geurts, Hanneke E. Hulst

**Affiliations:** 1 Department of Anatomy and Neurosciences, MS Center Amsterdam, Vrije Universiteit Amsterdam, Amsterdam Neuroscience, Amsterdam UMC VUmc, De Boelelaan 1117, Amsterdam, The Netherlands; 2 Anatomy and Neurosciences, Amsterdam UMC Location Vrije Universiteit Amsterdam, De Boelelaan 1117, Amsterdam, The Netherlands; 3 Amsterdam Neuroscience, Brain Imaging and Neurodegeneration, Amsterdam, The Netherlands; 4 Health, Medical and Neuropsychology Unit, Institute of Psychology, Leiden University, Leiden, The Netherlands

**Keywords:** Multiple sclerosis, Isolated cognitive impairment, Cognition, MRI, Cognitive rehabilitation

## Abstract

**Objectives:**

To study the frequency of isolated (i.e., single-domain) cognitive impairments, domain specific MRI correlates, and its longitudinal development in people with multiple sclerosis (PwMS).

**Methods:**

348 PwMS (mean age 48 ± 11 years, 67% female, 244RR/52SP/38PP) underwent neuropsychological testing (extended BRB-N) at baseline and at five-year follow-up. At baseline, structural MRI was acquired. Isolated cognitive impairment was defined as a Z-score of at least 1.5 SD below normative data in one domain only (processing speed, memory, executive functioning/working memory, and attention). Multi-domain cognitive impairment was defined as being affected in ≥ 2 domains, and cognitively preserved otherwise. For PwMS with isolated cognitive impairment, MRI correlates were explored using linear regression. Development of isolated cognitive impairment over time was evaluated based on reliable change index.

**Results:**

At baseline, 108 (31%) PwMS displayed isolated cognitive impairment, 148 (43%) PwMS displayed multi-domain cognitive impairment. Most PwMS with isolated cognitive impairment were impaired on executive functioning/working memory (EF/WM; N = 37), followed by processing speed (IPS; N = 25), memory (N = 23), and attention (N = 23). Isolated IPS impairment was explained by a model of cortical volume and fractional anisotropy (adj. R^2^ = 0.539, p < 0.001); memory by a model with cortical volume and hippocampal volume (adj. R^2^ = 0.493, p = 0.002); EF/WM and attention were not associated with any MRI measure. At follow-up, cognitive decline was present in 11/16 (69%) of PwMS with isolated IPS impairment at baseline. This percentage varied between 18 and 31% of PwMS with isolated cognitive impairment in domains other than IPS at baseline.

**Conclusion:**

Isolated cognitive impairment is frequently present in PwMS and can serve as a proxy for further decline, particularly when it concerns processing speed. Cortical and deep grey matter atrophy seem to play a pivotal role in isolated cognitive impairment. Timely detection and patient-tailored intervention, predominantly for IPS, may help to postpone further cognitive decline.

## Introduction

Approximately 50% of all people with multiple sclerosis (PwMS) will get impeded in their day-to-day functioning due to loss of cognitive abilities, and may experience problems at work and ultimately a reduced quality of life [1, 2]. Problems with information processing speed and visuospatial memory are often observed first, although deficits in executive functioning and attention are frequently present as well [3, 4].

Many studies that include cognitive outcomes use an average cognition score to investigate the relationship with underlying pathology, such as lesions or other MRI derived outcome measures [5–8]. Consequently, information about domain-specific test performances gets lost among the heterogeneous, averaged data, whilst this information may be relevant for the development of patient-tailored cognitive interventions. Also, averaged cognitive scores may disguise cognitive impairment since above-average scores on one cognitive domain may (partly) compensate for below-average scores in another cognitive domain. Impairment in one cognitive domain can already lead to problems in daily life, even if other cognitive functions are still intact [9]. Furthermore, isolated cognitive impairment has recently been found to have predictive value for cognitive decline over time, especially when impairment in memory functions or processing speed is present, further decline can be anticipated [4, 10]. This emphasizes the need for a better understanding of the underlying neurobiological correlates of specific cognitive impairments in PwMS.

Therefore, we first aimed to identify the frequency and distribution of isolated cognitive impairment across different cognitive domains in a cohort of long-standing multiple sclerosis patients. Second, we examined whether structural MRI measures can help to explain the neurobiological underpinnings of different forms of isolated cognitive impairment. Last, we evaluated the development of isolated cognitive impairment over time, trying to identify which PwMS are most susceptible to future (multi-domain) cognitive decline.

## Materials and methods

### Participants

PwMS who had been diagnosed according to the McDonald Criteria [11] as well as healthy controls (part of the Amsterdam Multiple Sclerosis Cohort) were included for MRI and neuropsychological evaluation [12, 13]. All patients had received a diagnosis of multiple sclerosis and were recruited from the Amsterdam MS Center when having a disease duration of 10 years since the first symptoms. Disease type was documented on the day of imaging. Patients were on different disease-monitoring drugs among which ß-interferons, glatiramer acetate, natalizumab, or other immunosuppressives. Former studies with the same dataset examined functional underpinnings of global cognitive impairment and the relation between average cognitive impairment and atrophy [14–16]. Inclusion criteria were disease duration of at least 10 years since the first appearance of symptoms. Exclusion criteria were relapses and steroid treatment in the two months prior to MRI and neuropsychological examination, as well as neurological and/or psychiatric comorbidities. The level of education was assessed on a scale from 1 to 7 (ranging from ‘not finished primary school’ to ‘acquired a university degree’) [17]. PwMS’ disability was measured using the Expanded Disability Status Scale (EDSS) [18]. Ethical permission for the study has been provided by the institutional ethics review board of the Amsterdam UMC, location VU University Medical Centre, and participants had given written informed consent prior to participation.

### Neuropsychological evaluation and composition of cognitive phenotypes

Neuropsychological evaluation was performed for all participants at baseline and at five year follow-up, and included an extended version of the Rao’s Brief Repeatable Battery of Neuropsychological Tests [19]. The test battery consisted of the Selective Reminding Test (SRT; verbal memory [20]), using the average scores of story recall, long term recall, consistent long term recall and delayed recall; Symbol Digit Modalities Test, using the total number of correctly written substituted combinations in 90 s (SDMT; information processing speed [21]); 10/36 Spatial Recall Test (SPART; visuospatial memory [19]), using direct recall average and delayed recall average; Word List Generation (WLG; verbal fluency [19]), using the total number of correct responses in 60 s on three trials: animals, professions, and words starting with the letter M; Stroop colour-word test (executive functioning—inhibition, cognitive flexibility; cards 1 and 2 for attention), the interference between the cards was expressed as time of card 3 minus the average of cards 1 and 2 [22–25]; Concept Shifting Test, using the ascending number ordering, alphabetical letter ordering and alternating letter and number ordering conditions (CST; executive functioning—cognitive flexibility [26]) and the Memory Comparison Test (per cent sign, one-, two-, three- and four-letter trials), with the difference between the time on the 4-letter trial and the 1-letter trial taken (MCT; working memory [27]). Raw test scores were corrected for age, sex and education using regression-based normative data (not-published).

Based on the cognitive constructs that were measured per sub-test, four cognitive domains were composed: information processing speed, visuospatial and verbal memory, executive functioning and working memory, and attention. An overview of which tests were used to define each of the domains is provided in Table 1. To generate an overall-score per domain, Z-scores of the sub scores per test within this domain were averaged. By generating domain-specific Z-scores, we are able to determine whether PwMS are cognitively deteriorating without intertwining test scores belonging to different cognitive domains. Next, domain scores of Z < − 1.5 compared to normative domain data were classified as ‘impaired’ [28, 29]. Domains with Z ≥ − 1.5 were classified as ‘preserved’. At baseline and follow-up, based on the Z-scores in each cognitive domain, patients who were impaired in a single domain were classified as having ‘isolated cognitive impairment’ and appointed to the eponymous phenotype, whilst PwMS who were impaired in two or more domains were appointed to the ‘multi-domain cognitive impairment’ phenotype. PwMs who were cognitively preserved on *all* domains were appointed to the ‘cognitively preserved’ phenotype. Subsequently, reliable change indices (RCI) were calculated to examine the development of cognitive phenotypes over time. RCI-values ± ≥ 1.64 were considered as reliable change [30], and were calculated by using the following formula:

**Table 1:** Overview of composed cognitive domains

Domain	Included sub-scores per test
Information processing speed	SDMT
Memory (visuospatial and verbal)	Verbal: SRT LTS first, SRT LTR sum, SRT CLTR sum, SRT delayed recall Visuospatial:* 10/36* SPART direct recall, SPART delayed recall
Executive functioning/working memory	Executive functioning: Stroop interference, CST shifting, WLG total Working memory: MCT slope
Attention	Stroop card 1 and 2, CST numbers, CST letters

*SDMT* symbol-digit modality test, *SRT* spatial recall test, *LTS* long term storage, *LTR* long term retrieval, *CLTR* cumulative long-term retrieval, *SPART* spatial attention and recall test, *CST* concept shifting task, *MCT* multiple comparison test, *WLG* word list generation task

$$RCI= \left\{\left[Subject(x)2-Subject(x)1\right]- \left[{\text{average}}(HC2)-{\text{average}}(HC1)\right]\right\}/SEd$$

In this formula, x is a single subject test score. Average (HC) is the mean of the normative data, and *SEd* is the standard error of the difference score for healthy controls’ timepoints 1 and 2. At follow-up, PwMS were classified according to the number of affected domains similar to the procedure at baseline. Next, PwMS were defined as cognitively declining when, at follow-up, they were impaired in more cognitive domains than they were at baseline; as cognitively stable when they were impaired in the same number of cognitive domains at follow-up as they were at baseline; and as cognitively improving when they switched from being impaired in ≥ 2 domains at baseline to isolated cognitive impairment at follow-up, or from isolated cognitive impairment at baseline to cognitively preserved at follow-up.

### MRI

All participants were scanned on a 3 T whole-body system (General Electric Signa HDxt), with an eight-channel phased-array head coil. The protocol included a 3D T_1_-weighted fast spoiled gradient echo sequence for segmentation and volumetric measurements (repetition time [TR] = 7.8 ms, echo time [TE] = 3 ms, inversion time [TI] = 450 ms, flip angle = 12°, 1.0 mm sagittal slices, 0.9 × 0.9 mm^2^ in-plane resolution), a 3D fluid attenuated inversion recovery (FLAIR) sequence for white matter lesion segmentation (TR = 8000 ms, TE = 125 ms, TI = 2350 ms, 1.2 mm sagittal slices, 0.98 × 0.98 mm^2^ in-plane resolution), a double inversion recovery sequence (TR = 8000, TE = 125, TI = 498/2100, sagittal 1.2 mm slices, 1.12 × 1.12 mm^2^ in-plane resolution) for (juxta)cortical and cerebellar lesion detection, and a 2D diffusion tensor imaging sequence for white matter integrity assessment, covering the entire brain (five volumes without directional weighting, i.e. b0 and 30 volumes with non-collinear diffusion gradients, echo planar imaging, b = 1000 s/mm^2^, TR = 13,000 ms, TE = 91 ms, flip angle = 90°, 53 contiguous axial slices of 2.4 mm, 2 × 2 mm^2^ in-plane resolution).

### Tissue integrity and white and grey matter lesions

An extensive overview of the image analysis steps that were performed is published in Eijlers et al. [14]. In brief, white matter lesions were automatically segmented on FLAIR images using k-nearest neighbour classification with tissue type priors [12]. Initial segmentations were manually checked for accuracy and the resulting masks were registered to the 3D-T_1_ images, where lesion filling was performed. For further volumetric analyses, SIENAX and FIRST were used (both part of FSL; version 5.0.4; http://fsl.fmrib.ox.ac.uk) [31, 32]. All volumes were normalized for head size using v-scaling derived by SIENAX. Cortical grey matter lesions were identified according to the consensus recommendations by the MAGNIMS group [33]. Cortical lesions were identified on DIR images as hyperintense areas compared to surrounding normal-appearing grey matter, of at least 3 mm^2^ in size. White matter integrity was expressed as fractional anisotropy (FA), and measured using the diffusion-weighted images, corrected for head movement and eddy current distortions using the tract-based spatial statistics pipeline, which is part of the FMRIB Diffusion Toolbox (part of FSL).

### Statistical analyses

All variables were inspected for normal distribution using Kolmogorov–Smirnov tests and histograms, and log-transformed if not normally distributed. Differences between groups in terms of demographics and imaging parameters were examined using multivariate general linear models for normally distributed variables, and Kruskal–Wallis and Mann–Whitney tests for non-normally distributed variables. Collinearity checks were performed using Pearson’s correlation coefficients. In case of correlations ≥ 0.7, the predictor that correlated to most other predictors was excluded, to prevent overfitting of the model. Then, for each cognitive domain, cognitive functioning was predicted from MRI measures using forward regression models. Predictors were preselected using univariate regression models corrected for age, sex and education. Only significant predictor candidates were used for further analyses. To examine which (baseline) demographic and imaging measures had the largest predictive value to determine further cognitive decline, logistic regression was performed using cognitive decline *vs.* cognitively stable as outcome variable. Used input variables (outcome measures) were age, sex, EDSS, cortical grey matter volume, thalamus volume, hippocampus volume, white matter lesion volume, and white matter integrity. Bonferroni-corrected values of *p* ≤ 0.05 were considered statistically significant. Analyses were performed using Statistical Package for Social Sciences (SPSS; Version 28, Chicago, IL, USA).

## Results

### Baseline cognitive performance

A total of 348 PwMS and 96 healthy controls were included. PwMS had an average disease duration from diagnosis of 11.6 years (SD = 6.9, ranging from 1 to 34), 256/348 (73.6%) of PwMS presented with some form of cognitive impairment. Of the PwMS with cognitive impairments, 108/256 (42.2%) were impaired on a single domain; the others (148/256; 58.8%) were cognitively impaired in ≥ 2 domains (see Table 2). 92/348 (26.4%) PwMS were classified as cognitively preserved.

**Table 2:** Demographics of the different domain groups

	Isolated IPS (N = 25)	Isolated memory (N = 23)	Isolated EF/WM (N = 37)	Isolated attention (N = 23)	Multi-domain cognitive impairment (N = 148)	Cognitively preserved (N = 92)	Healthy controls (N = 96)
Age	46.63 (9.95)	49.74 (9.46)	45.96 (12.79)	48.78 (8.33)	47.23 (10.33)	47.23 (10.34)	45.87 (10.45)
Disease duration	11.4 (6.35)	11.72 (8.02)	10.32 (6.70)	13.30 (6.66)	10.43 (7.30)	10.43 (5.92)	n/a
Male sex (%)	4 (16.0)^a,c^	13 (56.5)^d,e^	13 (35.1)	3 (13.0)^a,c^	54 (36.5)	27 (29.3)	40 (41.7)
Education†	5 [3–7]	6 [2–7]	4 [1–7]	4 [1–7]	5 [1–7]	6 [1–7]	6 [1–7]
MS type RR/SP/PP	16/5/2	11/4/4	27/6/3	16/4/3	102/27/15	72/6/9	n/a
EDSS^†^	3.0 [1.0–8.0]	3.0 [1.5–8.0]	3.0 [0.0–8.0]	3.5 [1.0–8.0]	3.5 [0.0–8.0]^c^	3.0 [0.0–7.5]	n/a

Data are mean ± standard deviation unless indicated by ^**†**^, then data are median [range]

*n/a* not applicable

^a^Significantly different from control group (*p* ≤ 0.05)

^b^Significantly different from cognitively preserved group (*p* ≤ 0.05)

^c^Significantly different from cognitively impaired group (*p* ≤ 0.05)

^d^Significantly different from information processing speed group (*p* ≤ 0.05)

^e^Significantly different from attention group (*p* ≤ 0.05)

PwMS who had isolated cognitive deficits were most often impaired in the executive functioning/working memory domain (N = 37; 34% of the total number of PwMS with isolated cognitive impairment); with PwMS being affected on inhibition (N = 11), or cognitive flexibility (N = 10); six PwMS were affected on working memory alone; 10 PwMS were affected on combinations of the above). A total of 25 PwMS were impaired on IPS, followed by attention (N = 23), and memory (N = 23).

A total of 148 patients (42.5% of the total number of included patients) were impaired on multiple cognitive domains. Of these 148 PwMS, 108 were affected on IPS (73.0%), 106 on executive functioning/working memory EF/WM (71.6%), 100 on memory (67.6%), and 55 on attention (37.2%). Most of the PwMS with cognitive impairment were impaired in two domains (N = 93), followed by three (N = 44) and four domains (N = 11).

There were no differences in demographics (i.e., age, sex, educational level) between the different phenotype groups, apart from more men being present in the memory impaired group than in the IPS and attention groups. In Table 2, an overview of the demographic and clinical data of the groups is provided.

All imaging measures were present for all PwMS and healthy controls, except for cortical lesion counts which were only available for 208 PwMS (60%) due to (un)availability of DIR sequences in a subset of the patients. Comparisons of the imaging measures between the different groups showed that PwMS in the multi-domain cognitive impairment phenotype have lower volumetric measures of cortical grey matter (*p* = 0.002), white matter (*p* = 0.001), lesion volume (*p* = 0.013), thalamus volume (*p* =  < 0.001), hippocampus volume (*p* =  < 0.001), and lower fractional anisotropy of the white matter (*p* = 0.008) when compared to PwMS in the CP group, and lower volumetric measures of cortical grey matter (*p* < 0.001), white matter (*p* < 0.001), thalamus (*p* < 0.001), hippocampus (*p* < 0.001), and lower fractional anisotropy of the white matter (*p* < 0.001) when compared to controls (see Table 3). There were no differences in MRI measures between the isolated cognitive impairment groups. All groups of PwMS with isolated cognitive impairment as well as multi-domain cognitive impairment showed lower white matter, thalamic, and hippocampal volume than the healthy controls. A detailed overview of the imaging measures and differences between phenotypes is provided in Table 3.

**Table 3:** Imaging measures of the different cognitive groups

	IPS (N = 25)	Memory (N = 23)	EF/WM (N = 37)	Attention (N = 23)	Multi-domain cognitive impairment (N = 148)	Cognitively preserved (N = 92)	HC (N = 96)
Cortical GMV (L)	0.75 (0.06)	0.74 (0.04)	0.76 (0.04)	0.77 (0.05)	0.73 (0.06)^a,b^	0.76 (0.05)	0.78 (0.05)
White matter vol (L)	0.67 (0.03)^a^	0.68 (0.04)	0.67 (0.03)^a^	0.67 (0.04)^a^	0.66 (0.04)^a,b^	0.68 (0.03)^a^	0.70 (0.03)
Lesion vol^A^	4.06 (0.4)	4.00 (0.48)	3.79 (0.39)^c^	3.96 (0.36)	4.08 (0.41)	3.90 (0.34)	n/a
Thalamus vol (mL)	17.89 (2.02)^a^	18.61 (2.18)^a^	19.05 (1.69)^c^	18.3 (1.96)^a^	17.41 (2.75)^a,b^	19.19 (1.68)^a^	20.64 (1.33)
Hippocampus vol (mL)	8.86 (1.53)^a^	9.22 (1.31)^a^	9.6 (1.21)^c^	9.45 (1.36)	8.76 (1.46)^a,b^	9.69 (1.15)	10.28 (0.97)
Total no. of cortical lesions^B^	16 [1–76]	10 [0–75]	7 [0–43]	11 [0–183]	14 [0–127]	6 [0–78]	n/a
Fractional anisotropy	0.39 (0.03)^a^	0.39 (0.03)^a^	0.4 (0.02)^a^	0.39 (0.02)^a^	0.39 (0.03)^a,b^	0.40 (0.02)^a^	0.42 (0.02)

Numbers are mean ± standard deviations, unless otherwise stated

*HC* healthy controls, *WM* white matter, *FA* fractional anisotropy, *GMV* grey matter volume

^A^Numbers are log-transformed

^B^Numbers are median [range]

^a^Significantly different from control group (*p* ≤ 0.05)

^b^Significantly different from cognitively preserved group (*p* ≤ 0.05)

^c^Significantly different from multi-domain cognitive impairment group (*p* ≤ 0.05)

### Predictors of isolated cognitive deficits

#### Information processing speed

In the univariate analysis, IPS was positively associated to cortical grey matter volume (std. ß = 0.386, *p* < 0.001), white matter volume (std. ß = 0.359, *p* < 0.001), thalamus volume (std. ß = 0.516, *p* < 0.001), hippocampus volume (std. ß = 0.449, *p* < 0.001), fractional anisotropy (std. ß = 0.433, *p* < 0.001), lesion volume (std. ß = − 0.373, *p* < 0.001), and number of cortical lesions (std. ß = − 0.360, *p* < 0.001). In the multivariate model, cortical grey matter volume, white matter volume, and fractional anisotropy were included as candidate predictors. The model was able to explain 53.9% of the total variance (F(2,20) = 13.882, *p* < 0.001, adj. R^2^ = 0.539). Cortical grey matter volume (std. ß = 0.43, *p* = 0.04) and fractional anisotropy (std. ß = 0.41, *p* = 0.05) remained as predictors.

#### Memory

Memory was positively associated to cortical grey matter volume (std. ß = 0.308, *p* < 0.001), white matter volume (std. ß = 0.267, *p* < 0.001), thalamus volume (std. ß = 0.402, *p* < 0.001), hippocampus volume (std. ß = 0.410, *p* < 0.001), fractional anisotropy (std. ß = 0.279, *p* < 0.001), T_1_ lesion volume (std. ß = − 0.287, *p* < 0.001), and number of cortical lesions (std. ß = − 0.260, *p* < 0.001). In the multivariate model, cortical grey matter volume, white matter volume, thalamus volume, and hippocampus volume were selected as candidate predictors. The regression model was able to explain 49.3% of the total variance in the data (F(2,15) = 9.250, *p* = 0.002, adj. R^2^ = 0.493), with cortical grey matter volume (std. ß = − 0.70, *p* = 0.001) and hippocampus volume (std. ß = 0.36, *p* = 0.05) retaining in the model.

#### Executive functioning/working memory

EF/WM was positively associated with cortical grey matter volume (std. ß = 0.209, *p* < 0.001), thalamus volume (std. ß = 0.297, *p* < 0.001), hippocampus volume (std. ß = 0.197, *p* < 0.001), and fractional anisotropy (std. ß = 0.263, *p* < 0.001). The multivariate model for EF/WM consisted of cortical grey matter volume, thalamus volume, hippocampus volume, fractional anisotropy, and white matter volume as candidate predictors. The model was not able to significantly predict EF/WM in the current dataset (F(1,31) = 3.261, *p* = 0.081, adj. R^2^ = 0.066).

#### Attention

Attention was positively associated with age (std. ß = − 0.215, *p* < 0.001), EDSS (std. ß = − 0.181, *p* = 0.001), and disease duration (std. ß = − 0.231, *p* < 0.001). The multivariate model was not significant, however.

### Longitudinal development of isolated cognitive deficits at baseline

Cognitive follow-up data was available from 240 PwMS (71%). A detailed overview of changes in cognitive profiles over time (mean follow-up duration of 4.9 ± 0.9 years) is shown in Table 4. Longitudinal assessment showed that, of the PwMS who were defined as IPS impaired at baseline, 69% (N = 11/16) deteriorated over time. The remaining 31% (N = 5) remained stable. None of the PwMS with isolated cognitive impairment at baseline improved over time. More than half of PwMS who were memory impaired at baseline (N = 9; 56%) remained stable over time, 31% (N = 5) deteriorated even further in memory function, and 13% (N = 2) improved. In the EF/WM phenotype, 46% (N = 10) of PwMS remained stable over time, and an equal number of PwMS either cognitively worsened or improved over time (both N = 7; 27%). Almost half of the group of PwMS who were impaired on the attention domain at baseline showed improvement over time (N = 7; 41%). PwMS in the attention group who remained stable (i.e., impaired in one cognitive domain) switched to either memory or EF/WM, but not IPS. From the PwMS who were cognitively preserved at baseline, 54% (N = 39) cognitively declined over time. Most of these PwMS deteriorated on memory (N = 10; 26%), EF/WM (N = 10; 26%), or attention (N = 8; 20%). Also, 23% (N = 9) showed deficits in multiple cognitive domains at follow-up, switching from CP to the multi-domain cognitive impairment group. Only one PwMS deteriorated solely on IPS. Of the PwMS who were cognitively impaired at baseline, 83.5% remained so. PwMS who improved over time (N = 16) mainly improved on attention (N = 6; 38%), followed by inhibition (part of EF/WM domain; N = 3; 19%), visuospatial memory (N = 3; 19%), working memory (N = 3; 19%), cognitive flexibility (N = 3; 19%), and verbal memory (N = 1; 6%).

**Table 4:** Longitudinal development of cognitive impairment in groups

Baseline	Cognitive impairment per domain at follow-up						
	FU IPS (%)	FU memory (%)	FU EF/WM (%)	FU attention (%)	FU multi-domain cognitive impairment (≥ 2 domains; %)	FU cognitively preserved (%)	Total decline over time (%)
Baseline IPS (N = 16)	5 (31)	0	0	0	11^a^ (69%)	0	11 (69)^a^
Baseline memory (N = 16)	0	9 (56)	0	0	5^a^ (31)	2 (13)	5 (31)^a^
Baseline EF/WM (N = 24)	1 (4)	1 (4)	9 (38)	0	7 (29)^a^	6 (25)	7 (29)^a^
Baseline attention (N = 17)	0	2 (12)	3 (18)	2 (12)	3 (18)^a^	7 (41)	3 (18)^a^
Baseline md-CI (N = 95)	4 (4)	4 (4)	3 (3)	2 (2)	80 (84)	2 (2)	80 (84)^a^
Baseline CP (N = 72)	2 (28)^a^	10 (14)^a^	10 14^a^	8** (**11)^a^	9 (13)^a^	33 (46)	39 (54)^a^
Total # patients follow-up (BL)	12 (16)	26 (16)	25 (24)	12 (17)	115 (95)	50 (72)	145/250 (60)^a^

Cross tabulation of patients with multiple sclerosis changing between cognitive domains over time. The rows denote the group distribution at baseline, whilst the columns denote the group distribution at follow-up

*IPS* information processing speed, *EF/WM* executive functioning/working memory, *md-CI* multi-domain cognitively impaired, *CP* cognitively preserved, *BL* baseline

^a^These patients are cognitively declining over time

#### Cognitively stable or improving versus cognitively declining PwMS

Of the 73 PwMS with isolated cognitive impairment at baseline, 26 declined in five years’ time (and thus converted to multi-domain cognitive impairment; see Table 4), whilst 32 PwMS remained stable and 15 PwMS showed cognitive improvement. A logistic regression model assessing the effect of different baseline MRI and demographic measures—candidate predictors were age, sex, EDSS, cortical grey matter volume, thalamus volume, and hippocampus volume—on the likelihood that PwMS having isolated cognitive deficits would cognitively decline over time did not provide a significant outcome (Χ^2^(7) = 10.882, *p* = 0.144), meaning there is no particular MRI measure predictive for further cognitive decline from isolated cognitive deficits.

## Discussion

Ten years after onset of MS, 73.6% of PwMS present with some form of cognitive impairment. In the current study, most PwMS were cognitively impaired in multiple domains (and therefore defined as multi-domain cognitively impaired) at baseline. Forty-two percent (N = 108) of the PwMS were impaired in a single cognitive domain (defined as having isolated cognitive impairment). It is especially the latter group that is of interest considering (early) cognitive rehabilitation and the development of targeted interventions, given the progressive character of isolated cognitive impairments over time.

Most PwMS with isolated cognitive impairment were impaired on EF/WM, followed by IPS, memory and attention domains. In this work, cognitive domains were defined a priori, based on test constructs, instead of retrospectively based on test outcomes. Nevertheless, the fraction of PwMS who were cognitively impaired on ≥ 2 domains was similar to that found in data-driven works [10, 34, 35]. Overall, our findings show that, coherent with recent literature, PwMS tend to cognitively deteriorate over time, from being preserved to isolated cognitive impairment to multi-domain cognitive impairment [4, 10].

Considering the predictors of isolated cognitive decline, lower cortical grey matter volume and worse white matter microstructural integrity lead to a larger chance of IPS deficits, which is consistent with earlier findings on MRI underpinnings of IPS [9, 36]. Furthermore, lower cortical grey matter and hippocampal volume were associated with memory problems, both of which were also considered key for memory functioning in the literature [37–39]. We were unable to identify MRI underpinnings of EF/WM and attention in the current dataset. This could be a consequence of these domains being constituted by four different test constructs (i.e., verbal fluency, cognitive flexibility, inhibition, and working memory), which are all expressions of executive functioning, albeit in different ways [40]. This may have affected the within-group variability to such an extent that predictive modelling did not yield significant results. Moreover, most of the PwMS in the multi-domain cognitive impairment group who were impaired on EF/WM, were only impaired on one of the four sub scores. As we have merged the domains of EF and WM, this may have provided a skewed view on the distribution of isolated cognitive impairments. This may also explain the finding that most PwMS were affected on EF/WM instead of IPS and memory, as described in the literature [4], and the relatively high number of PwMS who improved on EF/WM over time. Another explanation could be that EF/WM is not based on structural MRI underpinnings, but functional measures instead [41], and that EF disorders may be very difficult to pick up by neuropsychological tests [42].

In line with this, aiming to predict determining factors for further cognitive decline over time in PwMS who suffer from isolated impairment did not yield significant results. Former work showed that, when including all PwMS in the current sample, cortical grey matter volume has the largest predictive value for future cognitive decline [8]. However, this was analysed with averaged cognition measures and remains undetermined for individual cognitive domains.

Within the multi-domain cognitive impairment group, most of the included imaging parameters were a priori worse compared to PwMS suffering from isolated cognitive impairment and the entire CP and healthy control groups. This suggests that, the more neurodegeneration at baseline, the worse the cognitive state in PwMS – which has been extensively described in literature [10, 35, 43–45]. Domain-specific MRI differences between phenotype groups may have been underestimated in the current sample, as a consequence of the small sample sizes for each cognitive domain and potentially insufficient statistical power. Furthermore, although a specific literature-based cut-off value was used for cognitive decline, there remains a chance that PwMS who were in the cognitively preserved group were already cognitively affected by the disease. As such, the data should be interpreted with caution.

Evaluation of isolated cognitive impairment over time showed that PwMS who suffered from isolated IPS impairment were twice as likely to cognitively deteriorate over time than PwMS suffering from any other form of isolated cognitive impairment (69% vs. 18–31%). Our findings thereby support the literature, implicating that baseline IPS impairment serves as a good proxy for further cognitive decline [4, 46, 47]. Overall, PwMS in the memory group were relatively cognitively stable over time. An interesting finding in the longitudinal data was that PwMS who were in the attention group at baseline seemed to have the highest chance of switching to the CP group at follow-up. This potential could be leveraged by timely intervention, as was described in former works as well [48]. A connotation that should be placed here is that attention deficits may be mimicked by other factors e.g., sleep disturbances [2, 49]. Also, of the PwMS who had isolated EF/WM problems at baseline, the fraction of PwMS who remained cognitively stable or improved over time was markedly higher than in the IPS and memory phenotype groups. This is potentially due to the larger number of tests that patients had to simultaneously worsen on in order to decline on EF/WM.

This work is subject to some limitations. First, although our sample is large overall, the groups per isolated cognitive deficit are limited in sample size. As a consequence, we have merged the executive functioning and working memory domains into one group. This may have impeded specificity of the results. We have, however, chosen to include those cognitive domains that are mostly impaired in multiple sclerosis [1, 49]. Second, the current results should be interpreted with the connotation that PwMS in the examined cohort all had a disease duration of on average ten years. Therefore, external validity of the findings to PwMS with shorter or longer disease duration still has to be determined. Frequent monitoring of cognition, especially in early MS, is crucial as it might point towards more accurate predictors for early and further cognitive decline, and it provides intervention opportunities to postpone progression of cognitive impairment. Third, the number of predictors that were used as input for the regression model was relatively high and should therefore be interpreted cautiously. Also, cognitive domains were assessed using different numbers of tests. This may have influenced the results both in a negative and positive way, as chances of being classified wrongly are larger when classification is based on a single test. However, we do not consider the Z-scores a continuum in terms of treatment decisions: patients who are affected in any way should receive cognitive rehabilitation regardless of the precise measure of decline. Since there was no information on the extent to which patients were impeded in their day-to-day functioning, we were unable to shed light on further specific decline once patients were past a Z-score of ≤ − 2. Future works should take this into account. Last, the number of cortical lesions that were present was not available for all patients and their impact on isolated cognitive impairment might therefore have been underestimated in the prediction models.

The findings of this study highlight that isolated cognitive impairment is frequently present and can be a predictor for the development of (further) cognitive impairment in PwMS, emphasizing the need for timely assessment of cognitive performance in PwMS as was previously endorsed by the International MS and COGnition Society [2]. A brief assessment, for example with BICAMS [50], MACFIMS [51] or screening tools such as *Multiple Screener* [52], may offer a solution to identify cognitive impairment in its earliest phases, by identifying PwMS at risk for cognitive decline and providing early interventions. Consistent with previous literature, a reduced cortical grey matter and hippocampus volume, as well as changes in white matter integrity may play a role in the isolated impairments that seem most predicting for future cognitive decline. Future studies should work towards timely identification of cognitive impairment and the development of patient-tailored or cognitive domain-specific interventions, starting with the IPS domain, as particularly PwMS who are affected on IPS are prone to further decline over time.

## Data Availability

Anonymised data, not published in the article, will be shared upon reasonable request from a qualified investigator.
